# HPV Associated Head and Neck Cancer

**DOI:** 10.3390/cancers8080075

**Published:** 2016-08-05

**Authors:** Tara Spence, Jeff Bruce, Kenneth W. Yip, Fei-Fei Liu

**Affiliations:** 1 Department of Radiation Oncology, Princess Margaret Cancer Centre, University Health Network, Toronto, ON M5G 2M9, Canada; tara.spence@rmp.uhn.ca (T.S.); jeffpbruce@gmail.com (J.B.); ken.yip@utoronto.ca (K.W.Y.); 2 Department of Medical Biophysics, University of Toronto, Toronto, ON M5G 1L7, Canada; 3 Department of Radiation Oncology, University of Toronto, Toronto, ON M5T 1P5, Canada

**Keywords:** biomarker, head and neck cancer, head and neck squamous cell carcinoma, human papillomavirus, (HPV+), HPV negative (HPV−), microRNA, oropharyngeal carcinoma, prognosis

## Abstract

Head and neck cancers (HNCs) are a highly heterogeneous group of tumours that are associated with diverse clinical outcomes. Recent evidence has demonstrated that human papillomavirus (HPV) is involved in up to 25% of HNCs; particularly in the oropharyngeal carcinoma (OPC) subtype where it can account for up to 60% of such cases. HPVs are double-stranded DNA viruses that infect epithelial cells; numerous HPV subtypes, including 16, 18, 31, 33, and 35, drive epithelial cell transformation and tumourigenesis. HPV positive (HPV+) HNC represents a distinct molecular and clinical entity from HPV negative (HPV−) disease; the biological basis for which remains to be fully elucidated. HPV positivity is strongly correlated with a significantly superior outcome; indicating that such tumours should have a distinct management approach. This review focuses on the recent scientific and clinical investigation of HPV+ HNC. In particular, we discuss the importance of molecular and clinical evidence for defining the role of HPV in HNC, and the clinical impact of HPV status as a biomarker for HNC.

## 1. Introduction

Head and neck cancer (HNC) comprises a diverse group of tumours, with an incidence of over 500,000 cases annually worldwide [1]. The most common type of HNC is head and neck squamous cell carcinoma (HNSCC), the sixth most common cancer worldwide [2], which can arise from the oral cavity, nasal cavity, larynx, hypopharynx, and oropharynx. HNSCC pathogenesis has historically been associated with tobacco and alcohol use [3], but not all cases of HNSCC are associated with these high risk behaviours. The causative association between human papillomavirus (HPV) and cancer, a Nobel Prize winning discovery, was first recognized in the 1970s by Professor Harald zur Hausen, who identified the presence of HPV in cervical cancer [4]. Then, in 1983, evidence of HPV infection was identified in a subset of oropharyngeal carcinomas (OPC) [5]. Since that time, HPV has been clearly implicated as the causative agent in a subset of HNC [6]. Over the past 15 years, there has been a significant increase in the incidence of HPV positive (HPV+) HNC [7,8], particularly in tumours arising in the oropharynx (i.e., OPC), comprising approximately 60% of all cases [9,10,11]. This review summarizes the role of HPV in HNC, as well as the molecular, clinical, and demographic distinctions between HPV+ and HPV negative (HPV−) HNC. Furthermore, we discuss the importance of assessing HPV status as a clinically relevant biomarker, and therapeutic implications for the appropriate management of HPV+ HNC as a distinct diagnostic entity.

## 2. HPV and HNC

HPV is a non-enveloped, double-stranded DNA virus encoding a total of 8–9 proteins in approximately 8000 base pairs [12], with the ability to infect cutaneous or mucosal tissues [13]. There are approximately 179 distinct HPV genotypes [14], which can be divided into low risk and high risk groups based on their capacity to drive malignant transformation. The “high risk” HPV subtypes most clearly implicated in cancer are HPV16, 18, 31, 33, 35, 45, 51, 52, and 56, which are capable of causing cancers of the cervix, head and neck, anus, vagina, vulva, and penis [6]. The most commonly implicated subtype in HNSCC is HPV16, accounting for over 80% of HPV+ HNSCC [14,15]. Recently, our group examined HPV infection in 515 HNSCCs via RNA-sequencing data from The Cancer Genome Atlas (TCGA) [14]. Therein, we identified the presence of HPV transcripts in 73 (14%) of the HNSCCs, of which 61 (84%) were HPV16, and the other 12 (16%) were other subtypes (8 HPV33, 3 HPV35, 1 HPV56) [14]. Interestingly, HPV18, which is responsible for approximately 5% of cervical cancers [16], and which has been shown to contribute to approximately 2.5% of HNCs worldwide [15], was not identified in this cohort. Goodman et al. also recently reported the incidence of HPV subtypes in a cohort of 529 OPC samples, identifying HPV16 in 322 (61%) of the OPCs, and other high risk HPV subtypes in 56 (11%) of the OPCs, including: 31 HPV33, 14 HPV18, 11 HPV35, 4 HPV31, 4 HPV52, 3 HPV39, and 2 HPV45 [17].

**Figure 1:**
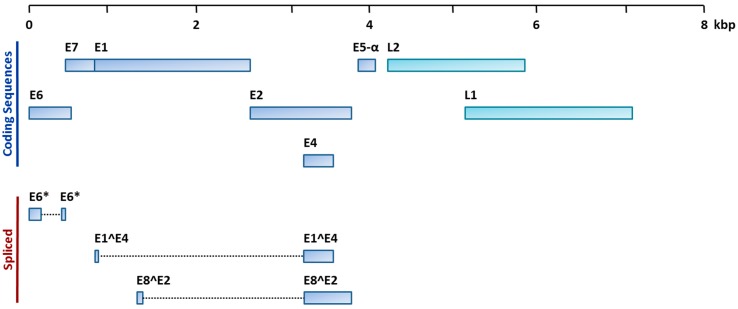
Structure of the HPV16 Genome (adapted from http://pave.niaid.nih.gov/, [12]). Coding regions and spliced transcripts encoded by the HPV16 genome are depicted. The early (E) genes encode the non-structural proteins that are produced early in the infectious cycle, while the late (L) genes encode structural capsid proteins that are produced late during viral infection.

The HPV capsid proteins L1 and L2 are responsible for tissue-specific infection. Replication is controlled by the E1 and E2 proteins, which regulate transcription of other viral genes [18]. A depiction of the HPV16 genome structure is shown in Figure 1. In HPV positive tumours, three viral proteins are responsible for oncogenesis; E5, E6, and E7 (reviewed in detail in [19]). The E6 and E7 proteins cooperatively inhibit apoptosis, promote uncontrolled cell proliferation, and induce genetic instability [20,21]. E6 activates the ubiquitin ligase E6AP, resulting in degradation of p53 [22], while E7 targets the retinoblastoma 1 protein (pRb) [23], causing de-repression of E2F, leading to overexpression of p16 and increased cell proliferation [24]. The function of E5 has yet to be fully elucidated; however, it is known to act together with E6 and E7 to promote transformation, and plays an important role in immune evasion [21,25].

## 3. Clinical and Demographic Features of HPV+ HNC

HPV+ HNC represents a distinct disease from HPV− tumours. Patients with HPV+ tumours present at a younger age (median = 57 vs. 64 years for HPV+ vs. HPV− OPC) [11], and are less likely to partake in excess alcohol consumption or heavy tobacco use [26]. A retrospective analysis of the annual trends in HNC from 1973–2003 demonstrated that although the annual incidence in smoking-related HNSCC has declined significantly over the recent decades at a rate of 1.85% annually, HPV-associated HNSCC has increased in incidence significantly, at a rate of 0.8% annually [27]. Additionally, HPV-related tumours more frequently arise in the oropharynx, whereas smoking-related tumours arise more commonly in the oral cavity, larynx, or hypopharynx [28]. HPV+ tumours are more likely to be smaller and poorly differentiated (with basaloid features), with a higher incidence of advanced lymph node (LN) metastases in comparison to HPV− tumours [29,30,31,32].

Despite a more aggressive clinical presentation, HPV status is the best independent predictor of survival in these patients [31,33,34]. An analysis of 493 HNSCC patients with LN metastases identified that HPV+ LN were larger and more likely to be cystic, yet they exhibited improved loco-regional control, whereby they regressed more quickly and were more likely to resolve following treatment in comparison to the LNs of HPV− patients [35]. HPV+ OPCs have a significant survival advantage over HPV− tumours, with a 58% reduction in mortality risk [34]. Additionally, HPV+ HNC patients with a history of tobacco use appear to fare worse than non-tobacco users with HPV+ HNC, indicating the prognostic importance of tobacco as a modulating factor in HNC [36,37]. Interestingly, the rate of distant metastasis (DM) is similar between HPV+ and HPV− OPC patients, and tumours of patients with HPV+ disease that develop DM appear to have a more aggressively disseminating phenotype [34,38]. However, the mechanism of DM development in HPV+ and HPV− patients has yet to be elucidated. Given the excellent loco-regional control of HPV+ patients, DM therefore remains as the major cause of death in these patients [11,38]. Methods to predict the risk of developing DM in HNC patients would enable refined risk stratification and inform appropriate treatment design for these patients.

## 4. Molecular Alterations in HPV+ vs. HPV− HNC

In both HPV+ and HPV− HNC, the p53 and pRb pathways are frequently altered; however, the mechanism of inactivation is distinct. As described above, the E6 and E7 viral proteins functionally inactivate p53 and pRb in HPV+ HNC. However, the majority of HPV− tumours have *p53* mutations, widespread copy-number loss, and promoter hyper-methylation and mutation of *CDKN2A*, leading to a loss of p16 expression [39,40]. Furthermore, genomic studies have identified distinct molecular differences between HPV+ and HPV− HNC, including divergent gene expression patterns, mutations, amplifications, and deletions [41,42,43]. HPV+ OPC frequently exhibit *TRAF3* loss, *PIK3CA* activating mutations, and *E2F1* amplification; while HPV− HNSCC commonly harbour 11q amplifications, and mutations in *CASP8* and *HRAS* [9]. However, despite these differences, HPV+ and HPV− tumours share frequent focal amplifications in 3q26/28, a region encoding the transcription factors *TP63* and *SOX2*, and the *PIK3CA* oncogene [9]. In addition, tumours harbouring integrated HPV exhibit differential patterns of DNA methylation, mutations, and gene expression as compared to episomal HPV+ tumours [39]. Transcripts expressed from an integrated virus are known to be more stable, and HPV integration is associated with a proliferative advantage [19], as well as increased genomic instability [44].

## 5. Determination of HPV Status and HPV as a Biomarker

HPV testing has recently been included in the American Joint Committee on Cancer (AJCC)/Union for International Cancer Control (UICC) guidelines as a standard pathological assessment for OPC [45]. However, guidelines for HPV detection have not been clearly defined, and methods for determining HPV status often vary between studies [46]. HPV detection by polymerase chain reaction (PCR) amplification of viral RNA from fresh or frozen tissues is widely accepted as the gold-standard method for determining HPV status [47]. To validate this and other detection methods in formalin-fixed paraffin-embedded (FFPE) tissues, our group examined three methods to determine HPV status: p16 immunohistochemistry (IHC), HPV16 (in situ hybridization; ISH), and quantitative real-time PCR (qRT-PCR) for HPV16 E6 mRNA. Though all three methods reliably detected HPV positive tissues, p16 IHC was more sensitive than HPV16 ISH, and is technically easier to perform than HPV16 E6 mRNA quantification by qRT-PCR [10]. We have now used this method extensively at the Princess Margaret Cancer Centre to confirm HPV positivity as a diagnostic marker, and facilitated the implementation of p16 IHC testing for OPC patients across multiple cancer centers in Canada [11,36]. However, it is important to note that although p16 is a reliable surrogate for determining HPV positivity, it cannot distinguish between HPV16 and other HPV subtypes. Methods for determining HPV status and distinguishing HPV subtypes in tumour tissues are summarized in Table 1.

### 5.1. MicroRNAs as Biomarkers for HPV+ HNC

MicroRNAs (miRNA) are small endogenous non-coding RNAs that are approximately 22 nucleotides in length [49]. Dysregulated miRNAs have established roles as both oncogenes and tumour suppressors [50], and act primarily through binding and direct repression of mRNA targets, thereby regulating gene expression [49]. MiRNAs have been proposed as useful tumour-specific biomarkers in cancer [51,52]. As such, a number of studies have examined the association between miRNAs and HNC prognosis. MiRNA signatures have been associated with HPV/p16 status, disease free and overall survival, and the risk of developing DM. Table 2 summarizes miRNAs implicated as putative biomarkers in HNC.

MiRNAs also hold potential as biomarkers for predicting treatment response in HNC, as a number of reports have shown the association between specific miRNAs with development of DM and survival (Table 2). Numerous studies have functionally characterized causal links between miRNAs and tumour development or progression in a subset of non-HPV related HNC (reviewed in [56]), which further highlights the utility of miRNAs as accurate tumour biomarkers. However, miRNA signatures have not been incorporated into clinical practice to date. Large-scale validation of existing prognostic and predictive miRNA signatures will be essential for their application as clinically informative biomarkers in HNC.

### 5.2. Circulating Biomarkers for HPV+ HNC

Circulating biomarkers indicative of HNC prognosis or treatment response would enable a non-invasive method of monitoring disease status and for improved treatment stratification. Few studies have identified circulating biomarkers for HNC; however, this important field of research is rapidly growing. Tinhofer et al. examined the persistence of circulating tumour cells (CTC) isolated from peripheral blood following treatment with adjuvant chemotherapy for locally advanced HNSCC. Interestingly, in the subset of OPCs within this dataset, CTCs were not predictive of disease free survival (DFS) or overall survival (OS). However, in non-OPCs, CTC were an independent prognostic marker of poorer DFS and OS [57]. Therefore, upon further validation, CTC quantification could prove to be a useful tool to identify patients who could benefit from treatment intensification.

Though outcome for HPV+ HNC patients is generally superior to HPV− patients, a subset of HPV+ patients have a poor prognosis, associated with the development of DM [11,58]. The ability to pre-identify the HPV+ patients with aggressive disease would facilitate optimal management of these patients in the clinic. A recent study examined the use of circulating neutrophil count (CNC), circulating monocyte count (CMC), and circulating lymphocyte count (CLC) as prognostic markers for HPV+ and HPV− OPC. The authors identified that high pretreatment CNC and CMC independently predicted for poor survival, and high CLC predicted for superior survival in HPV+ patients, whereas these trends did not correlate with survival in HPV− OPC [59]. This study therefore suggests the possible utility of pretreatment CNC, CMC, and CLC counts as early prognostic indicators for HPV+ OPC. Similarly, Wansom et al. examined the correlation between the level of circulating adaptive immune cells and outcome in HPV16+ OPC, finding that elevated pretreatment levels of CD8 T cells were associated with better response to induction chemotherapy, complete tumour response post chemoradiation treatment, and significantly improved survival [60]. Therefore, quantification of pretreatment CD8 T cell levels could provide valuable insight into the response to treatment for patients with HPV16+ OPC. Another study examined the level of HPV DNA isolated from the plasma of patients with HPV+ OPC. Pretreatment, HPV DNA was detectable in 65% of patients, and dropped rapidly to undetectable levels following radiation treatment [61]. In a subset of patients, HPV DNA increased to quantifiable levels at the time of DM development, highlighting the potential for this tool in detecting recurrence for patients with HPV+ OPC.

## 6. Therapeutic Perspectives: Treatment of HPV+ HNC as a Distinct Entity

Due to the distinct clinical and demographic features of HPV+ HNC as compared to HPV− HNC, HPV status has now been established as a reliable prognostic biomarker for this disease [36]. As such, patients with HPV+ HNC would likely benefit from unique disease management approaches [11,58]. Factors related to the inherent biological differences between HPV+ and HPV− tumours may influence the radiosensitive phenotype of HPV+ tumours. Studies have demonstrated that HPV+ tumour cells are intrinsically more radiosensitive than HPV− tumour cells, due to radiation-induced sustained cell cycle arrest [62,63]. This finding may be attributed to multiple factors, including decreased degradation of p53 by E6, resulting in re-activation of canonical p53 mediated cell cycle arrest and increased apoptosis [62], as well as impaired p16-mediated homologous recombination repair of double-stranded breaks [63]. Moreover, HPV+ tumours appear to be generally less hypoxic than HPV− tumours [64], resulting in a more radiosensitive phenotype, although there is conflicting evidence in this domain [65,66].

Smoking is well-implicated as a causative agent in HPV− HNC, and a history of heavy tobacco use is correlated with poor prognosis [28]. Smoking is also an important risk factor in HPV+ HNC [67]. Patients with HPV+ OPC with a history of smoking for more than 20 pack-years (PY) had a 2-year OS rate of 80%, as compared with an OS rate of 95% for HPV+ patients with a history of fewer than 20 PYs, though survival rates were still superior to HPV− OPC with over 20 PYs, with an OS rate of 63% [31]. Thus, tobacco exposure is a key prognostic indicator in both HPV+ and HPV− disease, and it is important to include smoking history within the criteria for disease risk stratification.

Immune modulation also appears to play a significant role in HPV+ HNC, which exhibit higher levels of immune infiltration than HPV− tumours [68]; these increased levels of immune infiltration in HPV+ tumours correlate with a significantly better outcome [69]. Furthermore, HPV16+ HNC patients have significantly higher levels of circulating T cells specific for HPV16 E7 protein, highlighting the essential role of immune modulation in HNC biology, as well as identifying a potential novel biomarker [70,71].

Current treatment regimens for HNC are aggressive and have significant treatment-associated toxicities, as a result of surgery, chemotherapy, and/or radiation treatment. These include both acute toxicities, such as mucositis, nausea, pain, hematologic changes, and stomatitis, among numerous other acute side effects, and late toxicities, such as chronic xerostomia, fibrosis, edema, trismus, and dysphagia [67]. Low risk HPV+ patients that are known to have significantly better survival rates would benefit from treatment de-intensification in order to minimize short and long-term treatment sequelae [72], while maintaining high rates of loco-regional control [73]. As such, a recent publication by our group described the need to refine the current recursive partitioning analysis (RPA) based TMN stage and prognostic groups for classification of HPV+ OPC, as defined by the AJCC/UICC classification system. The proposed criteria include patient age and smoking PY as well as the current RPA stage into four prognostic groups, yielding a significantly better prognostic performance than the current RPA classification [36]. Refining these disease risk classifications to identify low, medium, and high risk patients will undoubtedly improve HPV-associated OPC management, and allow for informed clinical trial design and appropriate selection of patients for treatment de-intensification trials. Chera et al. reported results of a Phase II chemoradiation therapy de-escalation trial for 43 patients with favourable risk, HPV+, and non-smoking associated OPC, with the primary endpoint of improved pathologic complete response (pCR). Encouragingly, patients experienced improved rates of pCR and decreased toxicity compared to standard therapies (NCT01530997, [74]). However, this study did not assess longer-term progression free survival (PFS), DFS rates, or late toxicities. The ECOG 1308 (NCT01084083) Phase II de-intensification trial examined PFS and OS of 62 HPV+ OPC patients who received low dose intensity-modulated radiation therapy (IMRT) with cetuximab following complete response to induction chemotherapy, as compared with 15 patients with partial response or stable disease who received standard dose IMRT with cetuximab. Preliminary findings of this trial report 1-year PFS rates of 91% and 87% for low dose and standard-dose groups, respectively [75]. Importantly, sub-group analysis of patients treated with low dose IMRT that had high risk features, including advanced tumour stage, nodal stage, and smoking history of over 10 PY, identified promising efficacy, with 1-year PFS rates of 86%, 88%, and 84%, respectively. Once long-term follow-up data is available for this trial, conclusions may be drawn regarding the effect of reduced IMRT dose on OS and late toxicities in HPV+ OPC patients. Another de-escalation trial was recently established by NRG Oncology and the National Cancer Institute (NCI)-US for patients with p16 positive, non-smoking associated OPC, to examine whether reduced IMRT doses with or without cisplatin treatment maintain PFS rates observed in patients treated at higher doses, while reducing treatment-associated toxicities (NCT02254278). Results from treatment de-escalation trials such as these will be essential to establish whether the current DFS and OS rates will be maintained following de-intensification of treatment for low risk HPV+ OPC.

### HPV Subtype Specific Treatment Considerations

As discussed previously, HPV16 is the most prevalent HPV subtype in HNC, contributing to over 80% of HNSCC [14,15]. Interestingly, patients with HPV16+ tumours appear to present at a slightly younger age than other HPV subtypes, with a median age at diagnosis of 56–57.5 years for HPV16+ vs. 59.5 years for other HPV subtypes [14,17], though the clinical importance of this difference remains unclear. Importantly, the OS rate for patients with tumours harbouring high risk HPV subtypes other than HPV16 is significantly lower than HPV16+ HNSCCs. Our group identified a 3-year OS rate of 88% for patients with HPV16+ HNSCC, while the 3-year OS was reduced to 49% for patients with the other HPV subtypes [14]. Similarly, Goodman et al. identified a 5-year OS rate of 65% for HPV16+ OPC, and a significantly worse OS rate of 46% for patients with other high risk HPV+ tumours, though HPV− patients fared worse, with a 5-year OS rate of only 28% [17]. These findings underscore an essential clinical distinction between HPV subtypes, and suggest that treatment de-intensification strategies might not be appropriate for all HPV+ subtypes.

## 7. Conclusions

In summary, HPV has become a recognized oncogenic driver in HNC, and the incidence of HPV+ HNC, particularly HPV+ OPC, is on the rise. HPV associated HNC is a distinct clinical entity, with significantly improved treatment response and survival rates in comparison to HPV− HNC. Clinical management of low risk HPV+ HNC may therefore require unique treatment approaches focused on de-intensification of current standard of care therapies. Results of new and ongoing treatment de-escalation trials in these low risk patients will be crucial for establishing whether the current rates of DFS and OS are maintained while treatment-associated toxicities are reduced.

HPV16 is the subtype most frequently associated with HNC, and current clinically relevant detection methods focus on identifying the presence of HPV16 by PCR or ISH, or detection of p16 protein expression in tumour samples by IHC. However, HPV+ tumours involving other HPV subtypes generally exhibit reduced survival rates, though these rates may be somewhat superior to HPV− disease. Thus, a subset of HPV+ tumours may not benefit from treatment de-intensification strategies, highlighting the importance of informed clinical trial design. Additionally, diagnostic and prognostic biomarkers, including HPV-associated miRNAs and circulating monocytes or lymphocytes, may prove to yield strong clinical utility, and warrant further investigation and clinical validation.

## Figures and Tables

**Table 1:** Methods for determination of HPV status and HPV subtype.

Method	Principle	Advantage	Disadvantage	Ref
**HPV16 E1 PCR**	HPV16, the most common HPV subtype implicated in HNC, is quantified by qRT-PCR in DNA extracted from bulk tumour tissue	Highly sensitive	False positives may occur; technically more difficult to perform than IHC/ISH; detects only HPV16	[10]
**p16 IHC**	p16 is upregulated indirectly via repression of pRb by E7; loss of p16 is common in HPV- HNC	Technically easy to perform and clinically feasible; comparatively low cost	Indirect method of HPV detection; does not distinguish between HPV subtypes	[10]
**HPV16 ISH**	HPV16, the most common HPV subtype implicated in HNC, is quantified and directly visualized in tumour cells	Technically easier to perform and clinically feasible; comparatively low cost; allows direct visualization of HPV in tumour nuclei	Detects only HPV16	[10]
**RNA-Seq**	Specific HPV viral transcripts can be detected by sequencing RNA transcripts	Accurate method for detecting HPV positivity and HPV subtype	High cost; technically difficult, requiring specialized resources; limited clinical feasibility at present	[14]
**DNA Sequencing**	HPV can be detected by DNA sequencing	Accurate method for detecting HPV positivity and HPV subtype	High cost; technically difficult, requiring specialized resources; limited clinical feasibility at present	[48]
**Roche Linear Array**	Detection of HPV by PCR amplification of DNA using HPV subtype specific primers	Accurate method for detecting HPV positivity and HPV subtype; most accurate method for resolving the presence of multiple HPV subtypes in one sample	Requires specialized resources; limited clinical feasibility at present	[48]

Abbreviations: head and neck cancer (HNC), human papillomavirus (HPV), immunohistochemistry (IHC), in situ hybridization (ISH), polymerase chain reaction (PCR), quantitative real-time polymerase chain reaction (qRT-PCR).

**Table 2:** MicroRNAs implicated as biomarkers in HPV+ HNC.

MicroRNAs	HNC Subtype	Expression in HNC ^¥^	Role as Biomarkers	Ref
miR-20b, miR-9, miR-9*	**OPC**	miR-9, miR-9* (up)	Associated with HPV/p16-status	[53]
		miR-20b (down)		
**miR-107, miR-151, miR-492**	**OPC**	miR-107, miR-151(up)	Correlated with overall survival	[53]
		miR-492 (down)		
**miR-20b, miR-107, miR-151, miR-182, miR-361**	**OPC**	miR-107, miR-151, miR-182, miR-361 (up)	Correlated with disease free survival	[53]
		miR-20b (down)		
**miR-151, miR-152, miR-324-5p, miR-361, miR-492**	**OPC**	miR-151, miR-324-5p, miR-361 (up)	Correlated with distant metastases	[53]
		miR-152, miR-492 (down)		
**let-7d, miR-205**	**HNSCC**	down	Associated with disease free and overall survival	[54]
**miR-210**	**HNC overall**	down	Associated with hypoxia; correlate with reduced overall and disease free survival	[55]

Abbreviations: head and neck cancer (HNC), head and neck squamous cell carcinoma (HNSCC), human papillomavirus (HPV), microRNA (miR), oropharyngeal carcinoma (OPC). ^¥^ Expression normalized relative to normal adjacent tissue [53], contralateral healthy mucosa [54], or histologically normal tonsil tissue from tonsillectomy patients [55].

## Abbreviations

The following abbreviations are used in this manuscript:

AJCC/UICC: American Joint Committee on Cancer/Union for International Cancer Control
CLC: circulating lymphocyte count
CMC: circulating monocyte count
CNC: circulating neutrophil count
CTC: circulating tumour cell
DFS: disease free survival
DM: distant metastases
FFPE: formalin-fixed paraffin-embedded
HNC: head and neck cancer
HNSCC: head and neck squamous cell carcinoma
HPV: human papillomavirus
HPV+: human papillomavirus positive
HPV−: human papillomavirus negative
IHC: immunohistochemistry
IMRT: intensity-modulated radiation therapy
ISH: in situ hybridization
LN: lymph node
miRNA: microRNA
NCIN: National Cancer Institute
OPC: oropharyngeal carcinoma
OS: overall survival
OSCC: oral squamous cell carcinoma
PCR: polymerase chain reaction
pCR: pathologic complete response
PFS: progression free survival
PY: pack years
pRb: retinoblastoma protein
qRT-PCR: quantitative real-time polymerase chain reaction
RPA: recursive partitioning analysis
TCGA: The Cancer Genome Atlas
